# First Surgeon to the Hospital of Versailles, on a kind of displacing of the Uterus, in the Case of eneysted Dropsy of the Ovarium and Fallopian Tubes, and on the Danger of wounding these Organs in those Operations which may become necessary

**Published:** 1804-01-01

**Authors:** M. Voisier


*Extract from a Memoir, presented to the Medical Society of Paris, by* M. Voisier, *First Surgeon to the Hospital of Versailles, on a kind of displacing of the Uterus, in the Case of encysted Dropsy of the Ovarium and Fallopian Tubes, and on the Danger of wounding these Organs in those Operations which may become necessary.*

EXAMPLES, without number, may be found in authors of encysted dropsy of the ovaria, but there is no where found instances of encysted dropsy of the Fallopian tubes. If, however, the existence of this disease may have appeared problematical, it is sufficient, in order to conceive the possibility, to recollect that this tube is capable, under particular circumstances, to become excessively enlarged, so as to contain a grown fœtus, with its appendages. Two memoirs, inserted in the Memoirs of the Academy of Surgery, Vol. 2. give an idea of the displacing of the uterus, in the case of an encysted dropsy of one of the ovaria; the first is by Monteulieu, jun. On opening the body, it was found that there was an encysted dropsy of this kind; that the uterus was more raised into the hypogastric region than usual, and inclined to the side of the diseased ovarium. In the second observation, published by Laporte, the uterus was found equally inclined to the side of the right ovarium, in which the disease was situated. The same displacing throughout takes place in the cases of encysted dropsy of the Fallopian tubes. It is only necessary to recollect the organization of these appendages, and their connexion with the uterus, in order to conceive that, in the case of a diseased enlargement of these parts, sufficiently great to fill the abdomen, they should directly act on the body of the uterus, by displacing it, and exposing it to be injured by instruments, used in the various operations which such cases require, either with a view to obtain a palliative or radical cure. The following is an unfortunate instance of the truth of this observation.—A woman, aged forty years, was brought to the Hospital of Versailles; her appearance was that of a person afflicted with ascites. The surgeon, a man of intelligence, performed the operation of the paracenthesis on the left side of the abdomen, at the usual place; four quarts of fluid came off in the first instance with considerable freedom; after which the water stopped suddenly; in vain a stilette was introduced through the canula, in order to remove the obstruction.—The pain which it produced, and faintings, ings, obliged the surgeon to withdraw it. The patient died three days after the operation. On opening the body, in the presence of the operator, there was found, instead of the right ovarium and Fallopian tube, a cyst sufficiently large to fill the entire abdomen, and which seemed formed by the enlargement of the coats and appendages of the uterus. This viscus was so raised in the abdomen above the superior margin of the pelvis, that it was pierced by the trocar in the operation. M. Dupuytren, to whom I communicated this observation, told me that he had frequently observed, in dead bodies, the kind of displacement in question. He has preserved three preparations illustrative of this fact. In this case the situation of the uterus is entirely deranged. The fundus uteri, dragged and raised by the cyst, inclines to that side the os tincæ is, therefore at the opposite side; and its edges, instead of being placed parallel to the symphisis of the sacrum and ossa ischia, become one superior, the other inferior. In this situation one of the sides corresponds to the junction of the sacrum with the vertebra, the other to the anteriour parietes of the hypogastrium. We conceive easily that this displacement may become still greater when a cyst, producing it, acquires a considerable volume; and when one of the Fallopian tubes contributes to form it, as we have seen in the case of the woman operated upon at Versailles. In this instance the uterus was placed in the abdomen, so as that one of its surfaces was glewed to the parietes of the cyst, and the other was turned to the lateral parietes of the belly, on the side opposite to the tumour. These observations may appear uninteresting to those who consider encysted dropsies of the abdomen as incurable, and the operations that are applicable, as useles, or even dangerous palliatives. It is the celebrated Dr. Hunter, (Medic. Obs. & Enq. vol. 2.) who favoured this opinion, although he was not ignorant that Dr. Bruheld had, in the year 1671, cured, by incision, an encysted dropsy, and which is accurately related in the Phils. Trans. 1724. Monro repeated, but unsuccessfully, this operation; but the person who has done the greatest honour to French surgery, the celebrated Le Dran, obtained a compleat cure of a dropsy of this kind, complicated with schirrosity, by means of an incision made into the most dependant part of the cyst, which became fistulous, and which was kept open for a long time, (Mem. de l'Acad. de Chirurg. tom. 2.)

M. Petit, Radel professor, relates that, in 1774, he radically cured a woman who had laboured two years under an ovarian dropsy, by means of the operation for the paracenthesis, and proper treatment. Encyclop. Method. Part. Chirur. tome ii. p. 132. Independent of these examples of radical cures, how many of palliative ones might be cited. I will confine myself to one observation by Sire-Jean, M. D. of Nancy. The girl, who was the subject of it, was submitted to the examination of all the physicians of that town. In consultation it was agreed that she had an encysted dropsy, but the kind could only be determined after death; the operation of the paracenthesis was performed fourteen times, and in consequence of which her life was preserved many years. But does there exist characteristic signs of encysted dropsies of the ovarium, and particularly of the Fallopian tubes, as well as of the displacement of the uterus, which they occasion? We must allow that the signs of this kind of dropsy described by authors, if they are considered separately, are rather equivocal; that the collateral circumstances which sometimes enable us to judge of their nature, may belong equally to a commencing extra uterine gestation. In the observation of Sire-Jean, we have seen that an entire college of physicians, although they suspected that the ovarium was the seat, and formed the cyst, confesses that he could not establish its real nature till after the death of the girl; this opinion is also confirmed by the 1223 Aphorism of Boerhaave, *Notabilis hydropis species quæ ovaria mulierum sæpe occupat difficulter cognoscitur et vix sine incisa cadavere.* Although it be very possible to confound an encysted dropsy of the Fallopian tubes with extra uterine pregnancy in this part, every practitioner will feel, that the fear of not distinguishing between these two cases cannot exist in the latter stages of this dropsy, where fluctuation, and the consequences of great compression, oblige us to have recourse to the operation of tapping. Besides, by a close examination of all the circumstances which precede and accompany the progress of the disease, we may know the seat of the disease in the affected side; but after what has been said, the displacement of the uterus necessarily existing in cases of dropsy of the ovaria and tubes, we may reckon the touch as the means most likely to throw light on the case. Thus then, in the case of encysted dropsy, suspected to depend on an affection of the ovarium and Fallopian tube, if to the symptoms common to all encysted dropsies of the abdomen, described in the works of the best authors, we find circumstances which prove that the affection began in the parts of generation, that its first devellopement vellopement was in the hypogastric region, where it had been preceded or accompanied by a painful sensation, should we not, in this case, look on this union of circumstances, when we add the absence of the uterus from the place it usually occupies in the pelvis, and the impossibility of reaching it with the touch, as a means of judging of the seat of the disease?

The following observation will serve to illustrate this assertion.

The widow C. 37 years of age, of a sanguineous temperament, addicted to violent passions, was delivered of her last child twelve years before; this birth was followed by a violent fever; during her convalescence she experienced in the left hypogastrium a painful sensation which did not afterwards quit her. A considerable irritation and pruritus about the labia produced excoriations which were taken for venereal, and the treatment which was the consequence rendered her situation still more unpleasant. From this period her belly swelled considerably, and became so voluminous that when consulted by her for the first time, in the eighth year of the Republic, I found her, to all appearance, labouring under ascites. The remedies usually employed in these cases were used without effect, and I determined to draw off the water by puncture. As the disease originally affected the genitals, and as she complained from the beginning of pain in the left side, which followed laborious parturition, the menses appearing regularly during the progress of the disease, but, particularly, as the swelling had begun in the left side, I suspected the existence of an encysted dropsy of the ovarium. With a view of ascertaining my suspicions, I had recourse to examination. In vain I sought after the os tincæ; I then determined to perform the operation on the left side of the abdomen. By means of the operation the cyst was completely emptied; the water that flowed was of the colour and consistence of ordinary urine. After the operation, by pressing on the left flank, a voluminous body was readily perceived; but, notwithstanding the evacuation of the waters, the neck of the uterus was imperceptible. During the months following this operation the patient felt better, but soon after the tumour filled again. The second operation was performed five months after, and near the first cicatrix. After evacuating the water, on withdrawing the canula, I placed in the cyst a small seton composed of a few threads; I recommended the patient to be constantly on the left side, in order to favour the evacuation of the water, water and prevent a new collection. The wound became as I wished, fistulous, and during eight months carried off constantly the waters as they were secreted. By persevering in this treatment, assisted by the use of aperients, tonics, and a suitable regimen, the woman became so far restored as to follow her usual occupations and even support the fatigue of a journey. Towards the beginning of the eleventh year the fistulous opening was allowed through negligence to close, on which the cyst began immediately to fill; I saw her towards the middle of last Brumaire (beginning of November.) The belly offered the appearance of an oblong tumour, extending itself from the left iliac region towards the umbilicus; I recommended the woman to undergo the same treatment which had already suceeded so well. She sent for me again at the end of four days; the tumor no longer existed, the water that formed it had been let off by an opening made by the patient herself with a bad penknife at the navel, and where the water had caused a considerable prominence; I recommended her to keep this open, and to observe the regimen already described. Towards the end of last Frimaire her situation got worse, and she died on the 8th of Nivose in consequence of an indigestion and a vomiting which it had occasioned. The following day the body was opened in the presence of my colleagues, M. Lamayran and Muhault, and the pupils of the hospital. On opening the abdomen the peritoneum was found every where connected to the viscera, the intestines were livid, and the omentum exhausted of its fat, and in many parts covered by a puriform matter. The uterus was raised into the hypogastrium above the superior margin of the pelvis, and inclined towards the left flank; the Fallopian tube of this side as well as the ovarium had entirely disappeared, but in their stead was observable a membraneous sac adhering firmly to the left side of the body of the uterus, and of which it seemed to make a part; the surface of this sac was thin, and in some places gangrenous, and extended to the navel, terminating at the small opening which the patient herself had made. This sac was narrower towards the navel, and larger towards the uterus, and occupied all the left lumbar and iliac regions, and contained about four ounces of a brownish flaky matter. The cavity of the matrix was, notwithstanding these appearances, perfect as well as the tube of the right side, but the ovarium was much enlarged, and contained many hydatids of the size of a filbert; without this increase of weight, which seemed to counterbalance the left side, it is probable probable that the displacing of the uterus would have been more considerable. We see from the appearances on inspecting the body of this woman, that the opinion that had been formed of the nature of her complaint was founded. If the uterus and its appendages were found so far displaced after death, how much greater must this have been before the first operation, when the tumor extended over a much greater surface of the abdomen?

It may be useful however to remark, that the means pointed out for ascertaining the nature and kind of dropsy in question, and the displacing which it causes, in order to avoid the danger of wounding it in operating, may be in some instances insufficient. In such a case, rather than run the danger of wounding, would it not be preferable to make the puncture at the navel rather than in the usual way?

The author concludes his Paper by arranging under four heads the reflections and observations which he has offered.

1. That encysted dropsy of the ovaria and Fallopian tubes may occasion the displacing of the uterus, and that in this case the uterus is raised out of the pelvis, and inclines from the side of the cyst which drags it.

2. That then the uterus is placed at the opposite side to that of the tumour, so that if it be the left ovarium and tube that form the cyst, the sac will be situated between the uterus occupying the right and left side of the integuments of the abdomen, and vice versa.

3. That if we have recourse to various operations used in such cases, without ascertaining the affected side, and the position of the uterus, we run a risk of wounding the uterus, and causing death.

4. That the means of avoiding this danger is to open the cyst on the side of the seat of the disease, or at the navel if the tumour extends to this part.

